# Repurposing GeneXpert® for rapid diagnosis of HPV-associated anal cancer: a case report of an unusually large lesion

**DOI:** 10.1186/s40249-026-01457-2

**Published:** 2026-06-01

**Authors:** Livia Bresciani, Jacqueline Concha-Jaimes, Alonso Díaz-Sheen, Jorge Miguel Sabina-Vela, Heidy Sanchez, Manuel Rojas-Blasquez, Kristopher Pinto, Paolo Vassalini, Cesar Johnny Ramal-Asayag

**Affiliations:** 1 Sapienza University of Rome, Rome, Italy; 2 Universidad Nacional Mayor de San Marcos, Lima, Peru; 3 Department of Infectious Diseases and Tropical Medicine, Hospital Regional de Loreto, Iquitos, Peru; 4 Department of Medical Oncology, Hospital Regional de Loreto, Iquitos, Peru; 5 Department of Radiology, Hospital Regional de Loreto, Iquitos, Peru; 6 School of Medicine, Universidad Nacional de La Amazonía Peruana (UNAP), Iquitos, Peru

**Keywords:** GeneXpert®, HPV-16, Anal cancer, HIV, Molecular diagnostics, Low-resource settings

## Abstract

**Background:**

Human papillomavirus (HPV)-associated anal squamous cell carcinoma (SCC) is strongly linked to persistent infection with high-risk HPV genotypes, particularly in people living with human immunodeficiency virus (HIV), who experience higher rates of viral persistence and oncogenic progression. The burden of HPV-related malignancies is disproportionately higher in low-resource settings, where limited access to screening, histopathology, and specialist care contributes to delayed diagnosis and advanced-stage presentation. In such contexts, rapid molecular diagnostic tools may help overcome diagnostic delays and support earlier clinical decision-making.

**Case presentation:**

We describe an atypically presenting HPV-associated anal squamous cell carcinoma in a man living with HIV from the Peruvian Amazon, a region characterized by geographic and structural barriers to healthcare access. The patient presented with a large exophytic perianal lesion measuring 16 × 7 × 4 cm^3^, exceeding sizes typically reported in the literature and reflecting prolonged disease progression. High-risk HPV type 16 was detected using a GeneXpert® assay applied to solid tissue, an approach rarely described, as most available evidence and current guidelines focus on swab-based specimens. Histopathological confirmation of HPV-associated squamous cell carcinoma was delayed due to limited local pathology services. In the interim, molecular HPV testing contributed to early diagnostic orientation and clinical planning. Imaging findings raised suspicion for advanced disease, although definitive metastatic staging could not be established due to incomplete diagnostic workup.

**Conclusions:**

This case highlights the potential utility of tissue-based GeneXpert HPV testing as a rapid adjunctive diagnostic tool in resource-limited settings where access to histopathology is delayed. Early molecular identification of high-risk HPV may support timely clinical decision-making and facilitate linkage to care. However, its clinical impact remains constrained in the context of advanced disease at presentation, underscoring the importance of addressing structural barriers to early diagnosis and sustained HIV and cancer care in underserved regions.

**Graphical Abstract:**

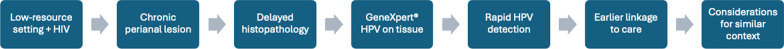

## Background

Among people living with human immunodeficiency virus (HIV), infection with human papillomavirus (HPV) is highly prevalent, with estimates exceeding 80%–90% even among individuals receiving effective antiretroviral therapy (ART). This population experiences increased rates of persistent infection, viral reactivation, and progression to malignancy, particularly anal squamous cell carcinoma (SCC) [1]. High-risk genotypes, especially HPV-16, account for the majority of oncogenic transformation. Although the risk is greatest in individuals with advanced immunosuppression, HPV-associated malignancies have also been observed in patients with relatively preserved immune function, including those with CD4 counts above the 200 cells/mm^3^ cut-off [2], suggesting that immune reconstitution alone may not fully mitigate oncogenic risk.

The natural history of HPV-related anal carcinogenesis involves progression from persistent infection to high-grade intraepithelial lesions and, in a minority of cases, to invasive carcinoma [3]. Early stages are often asymptomatic, contributing to delayed detection. Screening strategies—including anal cytology, high-risk HPV DNA testing, and high-resolution anoscopy—are recommended for at-risk populations [4,5]. However, access to these modalities is limited outside specialized centres and remains scarce in low-resource or geographically remote settings [6, 7]. Following screening, diagnostic confirmation typically requires histopathological evaluation of biopsy specimens, often complemented by imaging for staging. These processes can be time-consuming, resource-intensive, and not universally accessible, leading to delays in clinical decision-making and referral to specialized care.

In this context, approaches that streamline detection and diagnosis are particularly valuable. Rapid molecular assays to detect high-risk HPV, such as the GeneXpert® HPV test, offer a potential alternative by enabling timely detection of high-risk HPV DNA [8, 9]. In settings such as Peru, where GeneXpert platforms are already widely deployed for tuberculosis programs, existing infrastructure can be leveraged to expand access to HPV testing using relatively low-cost cartridges [10]. This integration may facilitate more efficient diagnostic pathways and improve access to molecular testing in resource-constrained settings [6, 7].

The burden of HPV-related cancers is disproportionately higher in low- and middle-income countries [11], largely due to insufficient access to prevention programs, early detection strategies, and specialized oncologic care. In regions such as the Peruvian Amazon, structural barriers—including geographic isolation, centralized healthcare systems, and limited availability of histopathological services—significantly hinder timely diagnosis [12]. Furthermore, cancer treatment services are not universally accessible, with many remote communities (such as riverine populations) effectively excluded from curative options, particularly when diagnoses are made at advanced stages [13].

Despite the availability of highly effective prophylactic vaccines, access to HPV vaccination remains uneven in many low- and middle-income countries. The World Health Organization recommends routine HPV vaccination for girls aged 9–14 years as a primary prevention strategy, ideally prior to sexual debut, with additional dosing considerations for immunocompromised individuals, including those living with HIV [14]. In Peru, HPV vaccination has been incorporated into the national immunization program since 2011 and is provided free of charge through school-based and community-based strategies, primarily targeting girls aged 9–13 years, with more recent expansion to include boys in the same age group [15]. Groups such as men who have sex with men (MSM)—especially those living with HIV—often depend on out-of-pocket access to preventive services, without targeted programmatic prioritization. And, whilst HIV treatment through antiretroviral therapy is provided free of charge in Peru, this alone is insufficient to address the broader structural and social barriers that continue to limit access to comprehensive prevention and care of HIV-related diseases [16].

Stigma represents a major additional barrier for access to healthcare for People living with HIV (PLWH), further increasing their vulnerability to infections to which they are already physiologically more susceptible. A recent study reported that 71% of PLWH in Peru experience HIV-related discrimination [17], and substantial evidence shows that stigma-related processes contribute to delayed healthcare seeking and lower engagement in prevention services [18, 19]. HIV-related stigma frequently overlaps with stigma toward sexual minorities—an effect widely documented in South American contexts, including Peru—creating a “layered stigma” that disproportionately affects MSM and transgender individuals [19]. This impact is substantial, particularly given that MSM represent a key population with a disproportionately high prevalence of HIV in Peru, with rates three times higher than those in the heterosexual population [19]. If we are to focus on the Peruvian Amazon, these challenges may be further exacerbated in certain communities, where access to information that could partially mitigate stigma-related barriers is more limited due to reduced exposure to education, media, and health campaigns. In these contexts, gaps in public knowledge and persistent misconceptions about HIV can contribute to stigma, social isolation, and avoidance of healthcare [16]. In this landscape, access to timely diagnostic confirmation represents a critical bottleneck.

This case report describes an atypically presenting HPV-associated anal SCC in an HIV-positive patient from the Peruvian Amazon, in which an unusually large lesion reflects prolonged disease progression and delayed access to care. It further aims to highlight the potential role of rapid, tissue-based HPV molecular testing as an adjunctive tool to support earlier diagnostic orientation and clinical decision-making in resource-limited settings.

## Case report: patient presentation

The case involves a male patient in his early 60s, with a known diagnosis of HIV infection and a reported stable adherence to antiretroviral therapy (ART) in the previous 20 years. Relevant background factors included MSM status and suboptimal living conditions characterized by lack of potable water and constant environmental exposure. The patient presented to our tertiary specialist unit in early April of 2025 with a progressively enlarging perianal lesion evolving over the previous 12 months. The lesion began as a ~ 1 cm wart-like growth, asymptomatic at first, later causing localized burning-type discomfort. At the time of presentation, the patient was receiving antiretroviral therapy with the once-daily fixed-dose combination of tenofovir disoproxil fumarate/ lamivudine/ dolutegravir (300/300/50 mg). Regional HIV program records documented multiple prolonged gaps (1–2 years) in ART pickup since diagnosis. The patient attributed these gaps to the difficulty of reaching the nearest ART distribution centre that for him required several days of travel by boat, limiting regular attendance.

Two weeks prior to admission the patient was empirically treated with intravenous antibiotics (ceftriaxone and metronidazole; exact dosage not documented) at a peripheral health centre. Only transient clinical improvement was observed, and the lesion continued to progress, prompting referral to the hospital for further assessment.

**Fig. 1:**
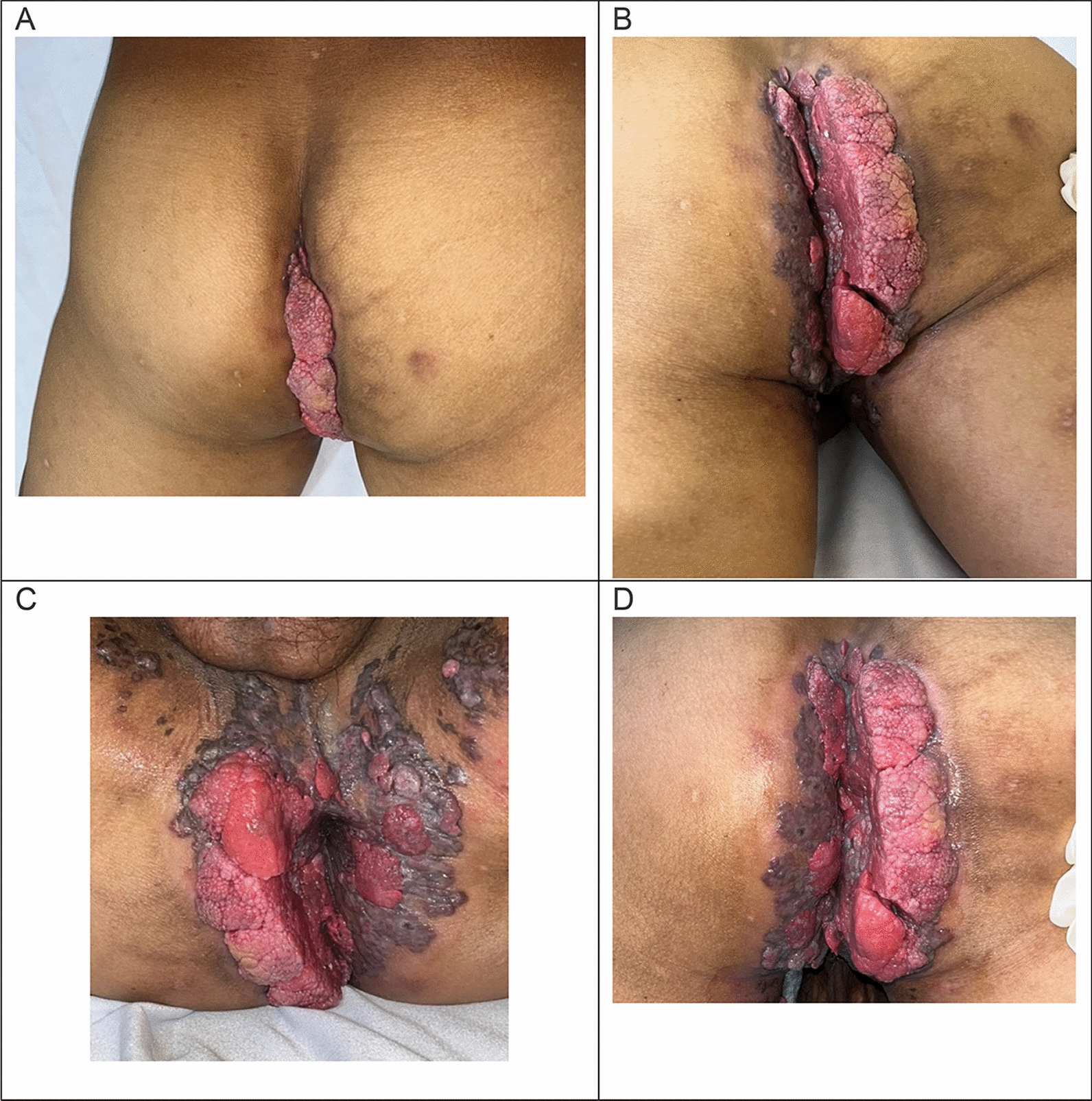
Clinical photographs of the perianal lesion at hospital admission (**A**) Posterior view in supine position demonstrating a large, exophytic, verrucous lesion arising from the perianal region and extending vertically along the intergluteal fold. (B) Close-up view showing the lesion’s irregular, lobulated surface with areas of ulceration, serosanguineous exudate, and surrounding perilesional hyperpigmentation, consistent with an advanced lesion

Upon admission, the patient denied systemic symptoms. On examination, a large externally visible, exophytic condylomatous lesion was noted involving the perianal skin, measuring approximately 17 × 13 cm^2^ on surface inspection (Fig. 1), with focal ulceration and serosanguineous, malodorous discharge. Bilateral inguinal lymph nodes were appreciated measuring approximately 1–2 cm, non-tender to palpation.

At baseline (day 1 of hospitalization), complete blood count showed a total leukocyte count of 7.72 × 10^3^/μl with slight absolute neutropenia. Slight normocytic anaemia was noted with a haemoglobin of 12.65 g/dL and haematocrit 36.9%. The biochemical profile revealed a mildly decreased albumin level of 3.11 g/dL (reference: 4.20–5.50), and an elevated alkaline phosphatase of 102.5 U/L (reference: 9.00–64.00). C-reactive protein (CRP) was slightly elevated at 0.54 mg/dL (reference: < 0.50). Renal and hepatic transaminases were within normal limits. The HIV viral load was undetectable (lower limit of detection: 40 copies/ml), and the CD4^+^ T-cell count was 323 cells/mm^3^, indicating preserved immune function, although on the lower end of the CD4 count spectrum. These viroimmunological data became available on day 14 of hospitalization.

## Case report: investigations and diagnosis

Within days six to 10 of hospitalization, viral serologies revealed non-reactive results for hepatitis A, hepatitis B and hepatitis C, syphilis treponema tests. Serological testing for herpes simplex virus typr-2 (HSV-2) showed positive immunoglobulin G (IgG) with negative IgM. Syphilis serology using rapid plasma reagin was also non-reactive. Three stool samples were analysed via microscopy and cultured, and were negative for intestinal parasites and Salmonella and Shigella.

Two elliptical skin biopsies measuring approximately 2 × 1 cm^2^ each were obtained from the perianal region and divided into approximately 0.5 cm sections for different analyses. Culture of the tissue sample of the perianal lesion yielded *Proteus mirabilis*. Antibiotic susceptibility testing was performed with interpretive standards consistent with Clinical and Laboratory Standards Institute (CLSI) guidelines. A multidrug-resistant profile was identified, susceptibility was retained towards meropenem (MIC ≤ 1 μg/ml).

Additionally, on day three of hospitalization, tissue samples were analysed using the Cepheid GeneXpert real-time PCR platform (Cepheid, Sunnyvale, CA, USA)[9]. This system is validated for liquid or semi-liquid specimens, so solid tissue samples therefore required pre-analytical processing prior to molecular testing. The processing approach was based on typical practices for the homogenization of solid tissue for molecular assays, adapted to the requirements of cartridge-based PCR platforms, and informed by local laboratory experience; tissue fragments were mechanically disrupted using sterile forceps or a mortar, and suspended in 3–4 ml of buffered saline to obtain a homogeneous solution, which maintains intra- and extra-cellular osmotic balance and preserves nucleic acid integrity during processing. The suspension was further macerated and mixed by brief vortex agitation (at least 10 s or 10–20 manual inversions) to ensure adequate cellular dispersion and nucleic acid release. After a short settling period of approximately 5 min to reduce gross debris, 2 mL of the homogenized suspension was carefully transferred into pathogen-specific GeneXpert cartridges, avoiding bubble formation. Cartridges were properly labelled prior to use, sealed, and loaded into the GeneXpert system to initiate the assay.

Search for *Mycobacterium tuberculosis* yielded negative results. Regarding HPV, the assay targeted conserved regions from 14 high-risk HPV genotypes, reporting HPV-16 individually, HPV-18/45 as a group, and the remaining 11 types (HPV-31, 33, 35, 39, 51, 52, 56, 58, 59, 66, 68) as a pooled result [9]. In our sample, HPV type 16 was detected, with no additional high-risk types identified.

Histopathological examination required five weeks to be completed (because of lack of specialist presence). In the interim, molecular HPV testing had already raised a strong suspicion of HPV-associated malignancy, which allowed early multidisciplinary planning, including surgical evaluation and consideration of additional therapeutic options, with early planning for referral to Lima should radiotherapy be required. This preparatory phase was particularly relevant given local constraints in access to specialist care and radiotherapy services. Additionally, the identification of high-risk HPV raised concern for malignancy and contributed to counselling the patient to remain hospitalized for further evaluation, despite his initial desire to return home.

Eventually, microscopic examination of the lesion confirmed SCC. Histopathological analysis demonstrated overexpression of p16INK4a (cyclin-dependent kinase inhibitor 2A, CDKN2A; hereafter p16), which we interpreted as strongly supportive of HPV-driven oncogenesis. Eventually, microscopic examination of the lesion confirmed SCC. p16 immunohistochemistry (IHC) was performed on formalin-fixed, paraffin-embedded tissue sections using the CINtec® p16 antibody (clone E6H4, Roche/Ventana, Tucson, AZ, USA). Staining was carried out according to routine institutional protocols. Interpretation followed standard criteria, with diffuse, strong nuclear and cytoplasmic (“block-type”) staining in tumour cells considered positive and consistent with HPV-associated oncogenesis.

**Fig. 2:**
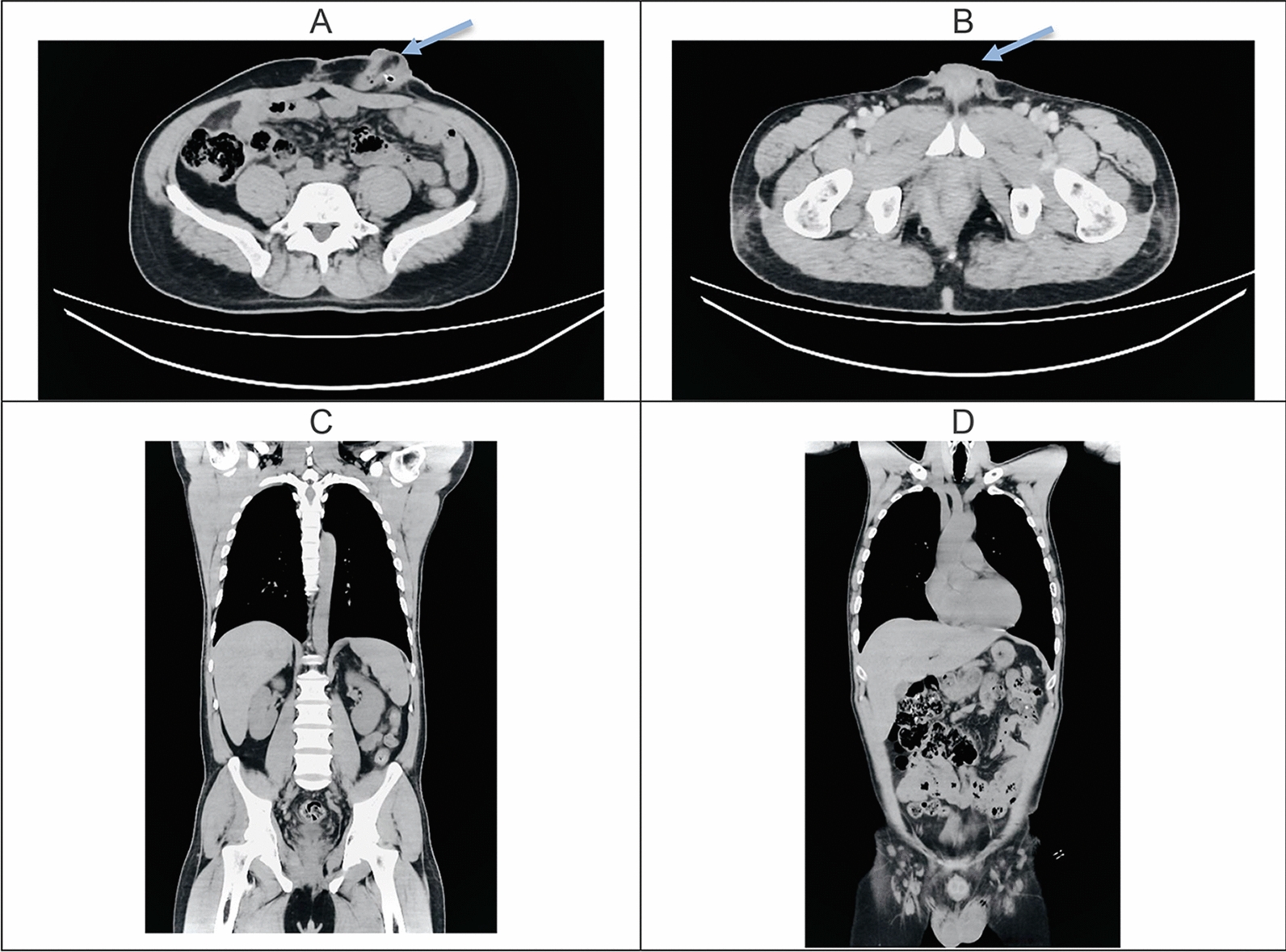
Axial images at the mid-abdomen (**A**) and pelvic levels (**B**); demonstrate a large, heterogeneous, exophytic soft-tissue mass arising from the perianal region, with irregular margins and areas of internal low attenuation suggestive of necrosis or debris (arrows). The lesion appears to extend superficially without clear invasion of deeper pelvic structures on these sections. Coronal reconstructions (**C**–**D**) show the craniocaudal extent of the lesion within the perineal region and confirm the absence of obvious intra-abdominal or thoracic metastatic disease

Contrast-enhanced abdominal computed tomography (CT), Fig. 2, demonstrated a solid heterogeneous mass measuring 51 × 23 mm^2^ in the right perianal region, representing the internal and deep tissue component of the lesion, distinct from its external portion observed on physical examination. No additional intra-abdominal lesions were identified. Based on CT findings, the lesion was assigned clinical stage IIb, most likely corresponding to cT3N0M0.

At week seven of hospitalization, a colonoscopy was not performed, as the multidisciplinary team—including gastroenterologists and oncologists—determined that the exophytic nature of the lesion would have rendered scope insertion technically challenging, with increased risk of bleeding and unnecessary patient discomfort. Furthermore, it would not have provided additional information for TN staging or therapeutic decision-making.

## Case report: treatment

Given the extent of disease, curative surgical resection was deemed unlikely to provide significant therapeutic benefit. Instead, a palliative surgical intervention was attempted: twenty-seven days after hospital admission, consisting in a loop colostomy at the level of the transverse colon. The tumour was confirmed intraoperatively as being confined to the distal rectum, without evidence of peritoneal carcinomatosis or gross metastatic disease. Regarding the isolation of *P. mirabilis*, targeted antimicrobial therapy with meropenem (1 g every 8 h intravenously) was administered for a total of 14 days. Following treatment, no significant improvement was observed in terms of discharge reduction or pain control. Based on the confirmed diagnosis of SCC and the extent of local disease, oncological treatment was determined in accordance with current guidelines [20, 21], consisting in combined chemoradiotherapy with mitomycin C and 5-fluorouracil, to be initiated after coordination of referral for out-of-region oncologic care. The patient remained clinically stable until discharge (after 42 days of hospitalization).

## Case report: follow-up

**Fig. 3:**
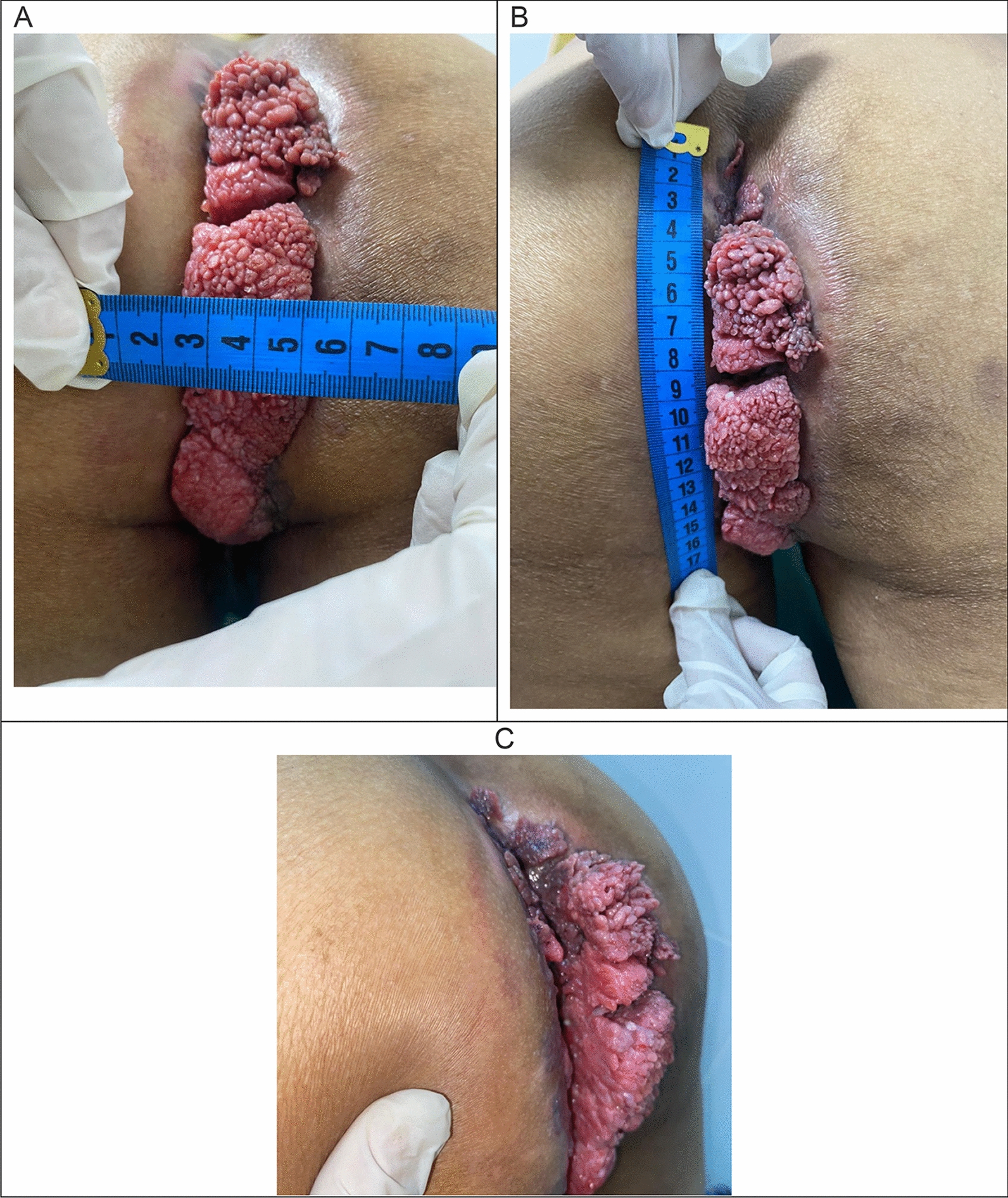
Sequential clinical photographs of the perianal lesion at follow-up on day 68. Top panel (**A** + **B**): Posterior view of the patient in supine position demonstrating a large, exophytic, verrucous lesion extending vertically from the perianal margin along the intergluteal fold, with a tape measure applied for reference. Bottom panel (**C**): Lateral view

At day 68 post-discharge, an ambulatory follow-up visit was performed.The lesion appeared clinically stable in size and characteristics (see Fig. 3). External measurements obtained using a measuring tape were approximately 16 × 7 × 4 cm^3^ (vertical, exophytic projection, and width, respectively). The patient reported subjective improvement, with better local hygiene and resolution of previously noted malodorous discharge.

Ultrasound revealed bilateral axillary lymphadenopathy with sonographic features suspicious for metastatic involvement. Although these findings could be compatible with stage IV disease, definitive metastatic staging could not be established. Further diagnostic workup was not performed because the patient subsequently disengaged from care and was lost to follow-up.

Selected published reports (Table 1) were identified through a targeted, non-systematic literature search. Studies were included based on clinical and methodological comparability to the present case, specifically: anogenital or perianal lesion location; large, exophytic, verrucous, or condylomatous morphology; histopathological diagnosis of squamous cell carcinoma; and availability of HPV-related diagnostic data, including histological assessment and/or molecular testing (e.g., PCR or p16 immunohistochemistry). Particular attention was given to reports describing large or “giant” lesions to contextualize the size of the lesion observed in our case; the included studies represent some of the largest lesions reported in the available literature.

**Table 1:** Selected published reports of large anal/perianal HPV-associated squamous lesions and squamous cell carcinoma relevant to the present case

First author	Patient demographics/HIV status	Lesion morphology/size; site	Histology	HPV testing method	HPV result	p16	Key relevance
Roy et al. (2025); [22]	Male, 40 y/o, HIV +	Giant fungating mass (~ 18 × 17 × 5 cm^3^); perianal	SCC	Tissue-based testing	HPV-6/11	Not specified	Closest size comparator; confirms HPV in very large lesions
Bastola et al. (2018); [23]	Male, 61 y/o, HIV +	Large exophytic mass (~ 15 × 10 cm^2^) with fistulae; Perianal/anorectal	BLT → SCC	Histology ± HPV context	HPV-associated (6/11 typical)	Not specified	Supports continuum: condyloma → SCC
Balogh et al. (2024); [24]	Female, 63 y/o, HIV -	Large verrucous (BLT) lesion; Anal/perianal	SCC transformation	Histology	HPV-related lesion	Not specified	Verrucous morphology comparator
Mix et al. (2023); [25]	Males, 34–75 y/o, HIV status not reported	Not size-focused; includes verrucous subtype; Scrotal	SCC (4 keratinizing, 1 verrucous)	PCR (37-type panel) + IHC	HPV16 (2 cases), HPV6 (1 case), 2 negative	p16 + in HPV16 cases	Demonstrates heterogeneous HPV involvement, including verrucous subtype HPV6-positive
Shenoy et al. (2019) [26]	Adults, HIV status not reported	Large condylomatous tumours; anal	SCC within BLT	Histology ± HPV	High-risk HPV association	Not specified	Supports malignant transformation pathway

SCC, squamous cell carcinoma; BLT, Buschke-Löwenstein tumour (giant condyloma acuminatum); HPV, human papillomavirus; HIV, human immunodeficiency virus; PCR, polymerase chain reaction; IHC, immunohistochemistry; p16, cyclin-dependent kinase inhibitor 2A (p16INK4a); MSM, men who have sex with men; y/o, years old

## Discussion

HPV-associated anal squamous cell carcinoma typically presents as an internally infiltrative lesion arising from the anal canal. The exceptionally large, predominantly exophytic perianal mass observed in this case reflects an uncommon presentation associated with an advanced local HPV disease burden. Indeed our summary of comparable reports highlight both the rarity of such lesions, with few lesions exceeding 5 cm reported. The application of GeneXpert-based PCR testing to solid tissue specimens has been rarely reported, particularly in the context of advanced HPV-associated anal disease.

In Peru, GeneXpert platforms are already deployed within the national tuberculosis control program, facilitating the use of existing molecular infrastructure for additional indications. In our case, this allowed molecular testing to be performed with costs largely limited to the HPV cartridge itself. The Xpert HPV assay is endorsed by the World Health Organization for primary cervical screening**,** owing to its standardized workflow, high sensitivity, and rapid turnaround time. However, this endorsement does not extend to anal cancer diagnosis or to routine testing of solid tissue specimens [27]. In cervical screening populations, Xpert HPV has demonstrated sensitivity of up to 90.8% for detection of cervical intraepithelial neoplasia (CIN) lesions above or equal to grade 2 [8]. In anal and perianal disease, high-risk HPV DNA testing—independent of platform—shows similarly high sensitivity but substantially lower specificity for clinically relevant lesions; pooled estimates indicate a sensitivity of approximately 90% for high-grade squamous intraepithelial lesion detection, with specificity of 47% in women and 35% in MSM living with HIV [8]. Thus, detection of high-risk HPV DNA alone does not establish malignancy, although it may support an HPV-driven carcinogenic pathway, given that the majority of anal squamous cell carcinomas are HPV-associated [8]. As the Xpert HPV assay is designed and validated for cervical specimens collected in liquid media, its application to solid tissue remains off-label, and performance characteristics in settings such as ours are still uncertain. It is evident that, molecular HPV assays are not sufficient as standalone tools for the identification of HPV related SCC, and are typically used alongside other diagnostic modalities, which can in any case prolong clinical decision-making. In our case, multiple approaches were required to support the diagnosis of HPV-related squamous cell carcinoma. The p16 biomarker was also evaluated; however, it did not expedite diagnosis, as results became available only after six weeks. Consistent with NCCN Anal Cancer Guidelines, p16 immunohistochemistry should not be used as a standalone surrogate marker of HPV-driven tumorigenesis, partly due to overexpression in non-HPV-driven tumours [28]. Together, these observations support the use of combined diagnostic approaches to improve specificity [29].

Looking ahead, there is a clear need for assays that are both rapid and highly specific. One promising example is mRNA-based testing to detect early genes E6 and E7. These genes are viral oncogenes that drive malignant transformation [14]. Their presence suggests a transcriptionally active infection, providing more clinically relevant information than DNA detection alone [8]. However, at present, even these mRNA-based assays are typically used in combination with HPV DNA testing or cytology, as part of established screening algorithms [14], to balance sensitivity and specificity and to ensure robust clinical validation across different populations [30].

In this case, definitive diagnosis was established at week six by histopathology. The patient was subsequently lost to follow-up at day 68, likely related to the prolonged time to diagnosis. The advanced stage at presentation, the delay in diagnosis, and the subsequent loss to follow-up all represent poor clinical outcomes. In keeping with the extent of disease, metastatic spread was suspected based on imaging showing bilateral axillary lymphadenopathy with features concerning for malignancy, following exclusion of common infectious causes. However, as axillary spread is atypical for anal squamous cell carcinoma, these findings were interpreted with caution. In line with current guidelines, lymphadenopathy identified on imaging requires tissue confirmation—such as fine-needle aspiration or biopsy—to establish definitive involvement [6, 16]. However, —regardless of confirmed metastasis—it remains important to understand why the disease progressed to such an advanced stage to better contextualize the role and limitations of HPV molecular diagnostics in similar settings.

Our patient resides in the Loreto region of Peru, which reports the country’s second-highest HIV incidence and the highest HIV-related mortality rate [31]. He demonstrated suboptimal immunological reconstitution, likely due to intermittent adherence to ART. Such fluctuations may have included periods with CD4 levels lower than at presentation, increasing vulnerability to persistent infections and adverse outcomes such as oncogenic progression. Furthermore, some of these infections are preventable through vaccination. Current international guidelines—including those for HIV-positive and other high-risk populations—recommend HPV vaccination primarily for preventing infection, even after the development of HPV-associated malignancy [7, 32]. In Peru, the HPV vaccine is provided free of charge only to adolescents aged 9 to 18 years [7]. Although our patient belonged to a recognized high-risk group (HIV-positive MSM), his age excluded him from eligibility under the public national immunization program; consequently, vaccination was not offered during adulthood following HIV diagnosis. This case highlights the need to evaluate the ethical and cost-benefit implications of extending HPV vaccination to high-risk adult populations, as implemented in several other countries [4].

With regards to case management and data provided: the lesion was not measured with a measuring tape (size only estimated) at baseline, which prevented precise assessment of tumour growth or dimensional changes during the 68-day follow-up evaluation. An additional limitation concerns the interpretation of the isolated *P. mirabilis* as a likely superinfection, given the lesion’s exophytic morphology, which is atypical of *P. mirabilis* infections [3] follow-up cultures could have clarified its contribution.

## Conclusion

This report highlights the potential clinical value of rapid HPV molecular testing in settings where access to anatomic pathology and specialist referral is delayed, such as the Peruvian Amazon. In this context, GeneXpert® HPV provided timely molecular evidence consistent with HPV-driven malignancy, supporting earlier oncologic decision-making and linkage to care in a system already constrained by geographic and logistical barriers.

## Data Availability

The patient’s medical record is archived at Hospital Regional de Loreto, Iquitos, Peru, and can be accessed upon reasonable request, in accordance with institutional policies and confidentiality regulations.

## Abbreviations

AIN: Anal intraepithelial neoplasia
ART: Antiretroviral therapy
BLT: Buschke-Löwenstein tumor
CD4: Cluster of differentiation 4 (T-helper lymphocytes)
CENARES: Centro Nacional de Abastecimiento de Recursos Estratégicos en Salud
CIN: Cervical intraepithelial neoplasia
CLSI: Clinical and Laboratory Standards Institute
CRP: C-reactive protein
CT: Computed tomography
DNA: Deoxyribonucleic acid
E6/E7: HPV oncogenes E6 and E7
ESMO: European Society for Medical Oncology
HIV: Human immunodeficiency virus
HPV: Human papillomavirus
HSV-2: Herpes simplex virus type 2
IHC: Immunohistochemistry
IgG: Immunoglobulin G
LMICs: Low- and middle-income countries
MIC: Minimum inhibitory concentration
MINSA: Ministerio de Salud (Peru)
MMWR: Morbidity and Mortality Weekly Report
MSM: Men who have sex with men
mRNA: Messenger ribonucleic acid
NCCN: National Comprehensive Cancer Network
PBS: Phosphate-buffered saline
PCR: Polymerase chain reaction
PLWH: People living with HIV
RPR: Rapid plasma regain
SCC: Squamous cell carcinoma
SIS: Seguro Integral de Salud
UNAP: Universidad Nacional de la Amazonía Peruana
WHO: World Health Organization
